# Food insecurity and associated factors during the COVID-19 pandemic in a vulnerable population in Rio de Janeiro: A primary care registry-based survey

**DOI:** 10.1371/journal.pgph.0005406

**Published:** 2025-12-15

**Authors:** Alessandra C. V. Meireles, Paula M. Luz, Daniel Csillag, Guilherme T. Goedert, Débora C. Pires, Emilia M. Jalil, Hugo Perazzo, Thiago S. Torres, Sandra W. Cardoso, Eduardo M. Peixoto, Beatriz Grinsztejn, Carlos A.M. Costa, Nadia C.P. Rodrigues, Breno Augusto Bormann de Souza Filho, Ana T.R. Vasconcelos, Rodrigo T. Amancio, Cleber V.B.D. Santos, Valdilea G. Veloso, Claudio J. Struchiner, Lara E. Coelho

**Affiliations:** 1 Escola Nacional de Saúde Pública, Fundação Oswaldo Cruz, Rio de Janeiro, Brazil; 2 Instituto Nacional de Infectologia Evandro Chagas, Fundação Oswaldo Cruz, Rio de Janeiro, Brazil; 3 Escola de Matemática Aplicada, Fundação Getulio Vargas, Rio de Janeiro, Brazil; 4 Instituto de Medicina Social Hesio Cordeiro, Universidade do Estado do Rio de Janeiro, Rio de Janeiro, Brazil; 5 Departamento de Saúde Coletiva, Universidade Federal do Rio Grande do Norte, Rio Grande do Norte, Brazil; 6 Laboratório de Bioinformática, Laboratório Nacional de Computação Científica (LNCC), Petrópolis, Brazil; 7 Hospital Federal dos Servidores do Estado, Rio de Janeiro, Brazil; 8 Department of Epidemiology of Microbial Diseases, Yale School of Public Health, New Haven, Connecticut, United States of America; Federal University of Bahia: Universidade Federal da Bahia, BRAZIL

## Abstract

Despite documented increases in food insecurity (FI) during the COVID-19 pandemic, the specific impact on highly vulnerable households within distinct urban contexts, as well as the interplay of various household-level factors in exacerbating this vulnerability, remain understudied. This study investigated FI prevalence and household-level factors associated with moderate/severe FI in *Complexo de Manguinhos*, a complex of slums in Rio de Janeiro, Brazil, during the COVID-19 pandemic. The Comvida-1 survey was conducted between September/2020 and February/2021 and included individuals aged ≥1 year. FI was measured using the Brazilian Food Insecurity Scale. Multiple Correspondence Analysis (MCA) identified associations between household variables (family composition, income, welfare benefits, job/income losses). Multilevel logistic regression assessed associations between the dimensions identified by the MCA and moderate/severe FI. Among 3864 households, moderate/severe FI prevalence was 14.7% (95% confidence interval [CI]: 13.5-15.9) and ranged from 4 to 28% across the 16 slums comprised in the *Complexo de Manguinhos*. Two MCA dimensions were associated with FI: Dimension 1 represented chronic socioeconomic vulnerability, with highest contributions from Bolsa Família benefit (10.73%), multiple children in Bolsa Família (9.56%), and presence of children in the household (6.84%). Dimension 4 captured acute COVID-19 economic impacts, including job/income loss (17.24%), reduced working hours (11.44%), and reduced family income (11.30%). Each dimension increased the odds of moderate/severe FI by approximately three-fold (Dimension 1 adjusted odds ratio [aOR]=2.72, 95%CI: 2.19-3.38; Dimension 4 aOR=3.32, 95%CI: 2.42-4.55, both p < 0.001). Both chronic socioeconomic vulnerability and acute COVID-19 economic shocks significantly predicted moderate/severe FI through distinct pathways. Dimension 1 reflected structural inequities from long-term poverty; Dimension 4 captured sudden economic instability. Effective interventions require layered approaches: strengthening social protections for chronic vulnerability (e.g., enhanced Bolsa Família benefits for families with children) while implementing rapid-response mechanisms for economic crises (e.g., emergency cash transfers and job retention programs).

## Introduction

The emergence of Coronavirus Disease 2019 (COVID-19) and the subsequent implementation of public health countermeasures to curb its transmission, such as physical distancing, quarantine, and lockdown, resulted in severe economic fallout that disproportionally affected the most unequal countries and vulnerable populations [1,2]. Work-post closures, layoffs, and reductions in working hours and wages resulted in increased unemployment and poverty [3–5]. Consequently, approximately 10% of the world’s population (about 702–828 million people) experienced hunger in 2021 [6].

The Food and Agriculture Organization of the United Nations defines food insecurity (FI) as the lack of regular access to enough, safe, and nutritious food necessary for normal growth and development and for maintaining an active and healthy life [7]. This concept encompasses not only the physical availability of food but also the access to it (economic, physical, and social resources to obtain food), its proper utilization (body’s ability to make use of nutrients), and the stability of these dimensions over time. FI manifests across a spectrum of severity, from mild (worry about food access) to moderate (compromised quality and variety, reduced quantities) to severe (experiencing hunger). The COVID-19 pandemic intensified these challenges through widespread economic disruptions as well as supply chain obstructions that hindered food distribution and increased food price volatility [8].

Beyond these macro-level drivers, household’s susceptibility to FI is influenced by household-level factors including the household’s income and employment stability, its composition (e.g., presence of children, elderly, or persons with disabilities), housing costs and stability, access to reliable transportation, and the overall health status of its members [9]. These factors often interact synergistically, creating unique vulnerabilities that determine a household’s resilience to economic shocks and its capacity to maintain consistent access to adequate food [8–10].

In Brazil, since 2004, FI has been regularly monitored by the Brazilian Institute of Geography and Statistics (IBGE) using the Brazilian Food Insecurity Scale (*Escala Brasileira de Insegurança Alimentar*, EBIA), with national surveys conducted in 2004, 2013 and 2018 [11]. During the COVID-19 pandemic, two national surveys, employing the same sampling design and methodology from IBGE, were conducted by the Brazilian Research Network on Food Sovereignty and Security (Rede PENSSAN), the first in December 2020 and the second in April 2022 [12,13]. Importantly, even prior to the COVID-19 pandemic, an increase in FI levels was documented in Brazil, a 38.1% rise from 2013 to 2018, and was deemed related to economic recession, fiscal austerity policies, and dismantlement of public policies to monitor, plan and fight hunger and FI [11]. The COVID-19 pandemic further aggravated this trend, and FI rose 55.2% from 2018 to 2020 and 72.2% from 2020 to 2022 [11].

Despite the well-documented global and national increases in FI during the COVID-19 pandemic, the specific and nuanced impacts on highly vulnerable households within distinct urban contexts, as well as the interplay of various household-level factors in exacerbating this vulnerability, remain an area in need of investigation. While national surveys provide broad epidemiological trends, they may not fully capture the unique experiences, specific drivers, and the differential impact of FI within marginalized communities. Therefore, this study investigated the prevalence and associated factors of FI among households in a highly vulnerable urban community in Rio de Janeiro, Brazil, during the COVID-19 pandemic. Our results shed light on the burden of FI in vulnerable urban settings and expand the understanding of chronic and acute socioeconomic stressors in relation to FI. These results could be used to guide public health policies and interventions such as the development of tailored food and nutrition policies that address the complex realities of life in vulnerable urban settings.

## Methods

### Ethics statement

All participants provided written informed consent before participating in the Comvida-1 study. For individuals under 18 years of age, parents or legal representatives provided consent. Legally incapacitated adults, according to Brazilian legislation, were not eligible for the study. Participants were clearly informed that participation in the study was entirely voluntary and of their right to decline participation or withdraw at any time without consequences. The study followed the Helsinki Declaration ethical principles for medical research involving human participants. Local ethics committees approved the study: Instituto Nacional de Infectologia Evandro Chagas (INI)/Fiocruz (CAAE # 3555558920.6.0000.5262), Escola Nacional de Saúde Pública (ENSP)/Fiocruz (CAAE # 35558920.6.3001.5240), and Instituto Oswaldo Cruz (IOC)/Fiocruz (CAAE #35558920.6.3002.5248).

### Study design and participants

The Comvida-1 study was a primary care registry-based cross-sectional serosurvey conducted in *Complexo de Manguinhos,* Rio de Janeiro, Brazil, to estimate the prevalence of anti-SARS-CoV-2 antibodies between September 15^th^, 2020, through February 10^th^, 2021. The study design and results have been published elsewhere [14].

Briefly, Manguinhos is a neighborhood located in the Northern area of Rio de Janeiro city, mainly comprised of slums, and has the 5^th^ worst Human Development Index (HDI) in the city [15]. According to the 2022 census, Manguinhos had an estimated population of 28,855 inhabitants and approximately 12,130 households [16]. The Manguinhos population is served by two public primary health clinics (*Clínica da Família Victor Valla* and *Centro de Saúde Escola Germano Sinval Faria*) which operate under the Family Health Strategy (*Programa de Saúde da Família*) of the Brazilian Unified Health System (*Sistema Único de Saúde, SUS*). These clinics maintain a regularly updated registry of the population for their catchment area, a process facilitated by the Community Health Agents. As the Family Health Strategy mandates comprehensive household registration within its coverage area, this registry provides a population enumeration and served as our sampling frame. Community Health Agents, who are both residents of the area and members of the primary care team, are responsible for home visits, registry upkeep, and various educational health promotion activities [17].

Individuals aged ≥1 years old residing in Manguinhos and registered in one of the two primary health care clinics listed above were eligible for sampling. Random sampling without replacement was employed. Sample size calculations were based on the team’s weekly field capacity for interviews and sample collection and the estimated prevalence of anti-SARS-CoV-2 antibodies. Details of the sampling procedures including the challenges encountered (harsh weather and violence) leading to inclusion failures have been published along with the primary outcomes of the *Comvida-1 study* [14]. From the parent Comvida-1 study, we included all participants who answered the FI scale as part of the study’s instrument (detailed below), S1 Table provides a comparison between those included and excluded from the present analysis.

#### Data collection.

A trained study team comprising two interviewers and one laboratory technician visited the address and invited the individuals to participate. After providing written informed consent, participants were interviewed face-to-face at home using a structured questionnaire administered via REDCap software on mobile devices. The instrument captured both individual-level variables (sex, age, education level, race/skin color, comorbidities, among others) and household-level variables (family income, household-level food insecurity, among others). For participants aged <18 years, the household-level data was provided by a primary adult (a parent or legal representative).

For this analysis we focused on household-levels variables, including monthly family income (measured in the number of minimum wages; minimum wage was 1,045 Brazilian Reais [BRL] in 2020, which corresponds to approximately 199 United States Dollars [USD]) [18], any family member participation in any governmentally funded beneficiary program (e.g., meaning if any household member was a beneficiary of the *Bolsa Família* or *Auxílio Financeiro Emergencial* cash transfer programs). *Bolsa Família* is a nationwide conditional cash transfer program for families in extreme poverty instituted in 2004 [19,20]. *Auxílio Financeiro Emergencial* was an emergency financial assistance program implemented during the COVID-19 pandemic (Apr 2020 through Oct 2021) targeting low-income informal workers, the self-employed, and those already registered in *Bolsa Família* who were eligible to receive this transfer in place of their regular *Bolsa Família* benefit [21,22].

Participants also answered the following questions: Since the beginning of the COVID-19 pandemic, has anyone in your household: (i) received food donation (yes/no); (ii) had their working hours reduced (yes/no); (iii) lost their job or any income source (yes/no).

Household composition (household size and age of household members) and household characteristics (which slum the household was located in, and the number of bedrooms) were also assessed. For the analysis, household composition information was used to define the following variables: presence of children (age 1–9 years) (yes/no); presence of adolescents (age 10–17 years) (yes/no); presence of elders aged ≥60 years (yes/no). Household density was calculated as persons per bedroom (number of household members divided by number of bedrooms), and later categorized as ≤1, 1.1-1.5, 1.6-3.0 and >3.0.

#### Outcome definition - Food insecurity.

FI was assessed using the EBIA scale, a 14-items validated scale that expresses the families’ perceptions regarding access to food [23,24] (S2 Table). Each affirmative answer (“yes” response) to any of the 14 items receives one point, while “no” and “don’t know” responses receive zero points, resulting in a score ranging from 0 to 14 points. This score enables the classification of households into food secure (0 points) and food insecure (≥ 1 point). Moreover, it classifies households into three levels of FI: mild (1–5 points), moderate (6–9 points) and severe (10–14 points). As in prior work, we have created a binary (dummy) variable named “moderate/severe FI”, in which food secure and mild food insecure households where grouped in a single stratum, while moderate and sever food insecurity composed the other stratum (EBIA ≥6 points) [25].

#### Statistical analysis.

We described the characteristics of the study population using frequency and percentages for categorical variables. Percentages comparison among the four groups defined by the EBIA scale (i.e., food security and FI mild, moderate and severe) was made using the chi-squared test. Prevalence of moderate/severe FI (EBIA score ≥ 6 points, yes or no) (with 95% confidence interval [CI]) was estimated using Poisson regression models, overall and for each one of the 16 slums comprised in *Complexo de Manguinhos*.

Multiple Correspondence Analysis (MCA) was used to identify the associations between independent household-level variables. “Moderate/severe FI” and “slums” variables were not included in the MCA. MCA can be seen as a generalization of principal component analysis when the variables to be analyzed are categorical instead of quantitative [26], it reduces dimensions while preserving relationships. MCA identifies the most important variables (and categories within the variables) that contribute the most in explaining the variations in the dataset. For all the variables included in the MCA, missing values were imputed using the modal category of each variable, that is, the most frequent category was selected.

Multilevel logistic regression models were used to assess the association of MCA’s dimensions with household moderate/severe FI (EBIA score ≥ 6 points, yes/no). To account for spatial clustering of participants in each one of the 16 slums comprised in *Complexo de Manguinhos*, we assumed varying intercepts, meaning that each slum would have a unique intercept, while assuming fixed effects for all other covariates, in this case, each MCA dimension. For the regression analysis, study data was randomly split into two mutually exclusive subsets: *training* (70%) and *testing* (30%). To evaluate the performance of models on the test subset, we considered a comprehensive set of metrics to address the different aspects of the classification performance, particularly due to the imbalanced nature of the outcome (food insecurity). The area under the receiver operating characteristic curve (AUC) was used as a measure of the overall discriminative power. Furthermore, we reported specificity and sensitivity to evaluate the models’ performance across both outcome classes. Accuracy is also reported for its intuitive value but was interpreted with caution, as it can be artificially inflated by the majority class.

Finally, we interpreted the MCA dimensions that were identified as significant predictors of moderate/severe FI in the regression models. We evaluated the most influential categories for each dimension by examining their contributions (measured as percentages, summing to 100%), with higher contributions indicating greater importance in defining the dimension. We also assessed category coordinates within each dimension (positive or negative directions) to determine how strongly each category is positioned along the dimension. Categories that cluster together indicate associations. Additionally, we visually explored moderate/severe FI prevalence for the most influential variables identified in the MCA dimensions.

MCA was perfomed using FactoMineR (for the analysis) and factoextra (for data visualization) packages; multilevel regression models were performed using lme4 package, all analysis were performed in R version 4.4.2 [27–29].

## Results

**Fig 1:**
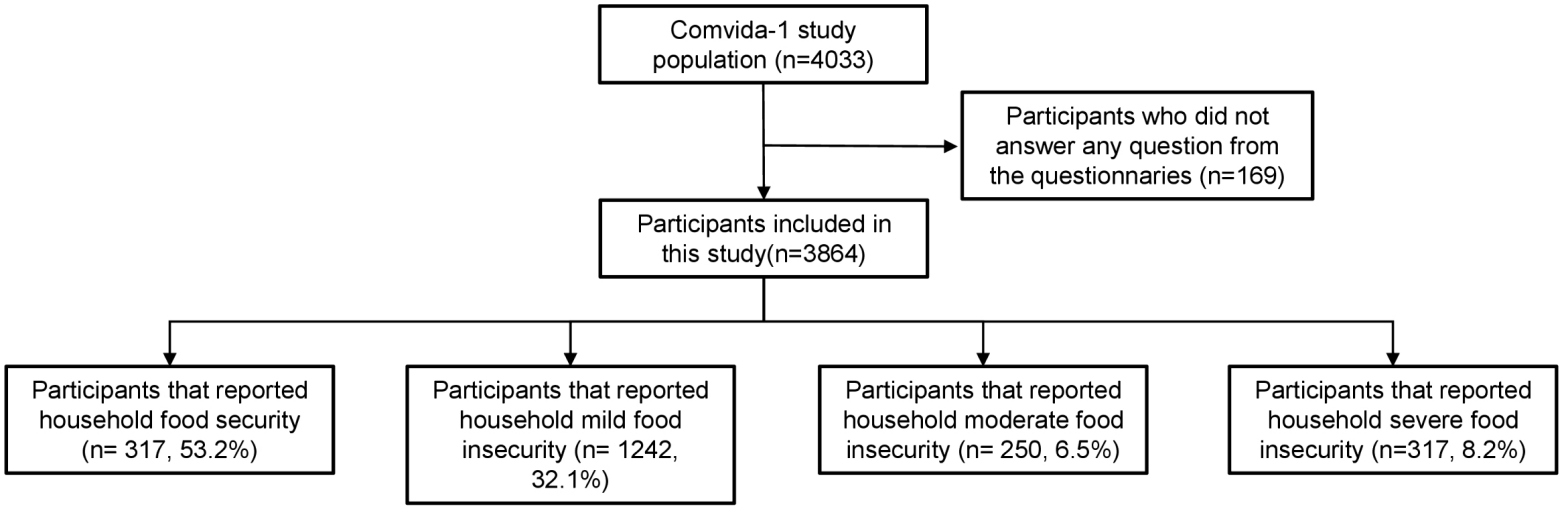
Flow chart of participant inclusion/exclusion and breakdown according to the score of the Brazilian Food Insecurity Scale (EBIA), the *Comvida-1* Study, Rio de Janeiro, Brazil, from September 2020 to February 2021 (n = 3,864).

The study population included 3,864 participants from the Comvida-1 study who completed the EBIA scale (Fig 1). Regarding age and sex, these participants were not statistically different from those included in the Comvida-1 parent study (S1 Table). Over half of the participants (53.2%) were food secure, whereas 32.1% experienced mild FI, 6.5% moderate FI, and 8.2% severe FI (Fig 1).

**Fig 2:**
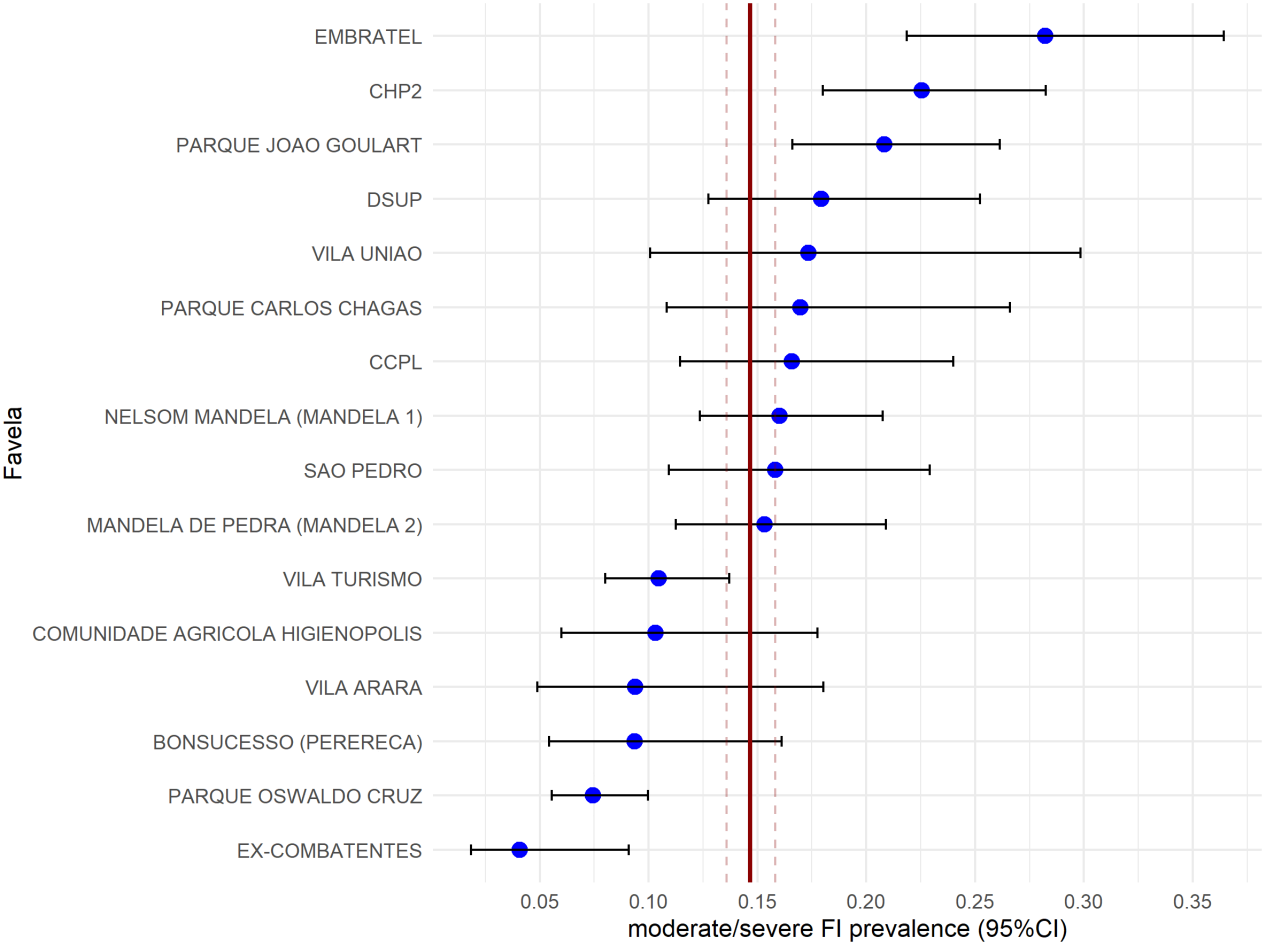
Prevalence of moderate/severe food insecurity (measured by the Brazilian Food Insecurity Scale, EBIA) by slum of the *Complexo de Manguinhos*, the *Comvida-1* Study, Rio de Janeiro, Brazil, from September 2020 to February 2021 (n = 3,864). Prevalence (blue dots) and 95% confidence intervals (horizontal black bars) estimated using Poisson regression models. The vertical red lines show the overall prevalence (solid) and 95% confidence intervals (dashed), 14.7% (IC95% 13.5-15.9). CPH2: Centro Habitacional Provisório 2, DSUP: Condomínio DSUP – Departamento de Suprimentos do Exército, and CCPL: Cooperativa Central dos Produtores de Leite.

The overall prevalence of moderate/severe FI across the study population was 14.7% (95% CI: 13.5-15.9), as illustrated in Fig 2. This figure further highlights the variability in the prevalence of moderate/severe FI across the 16 slums comprising *Complexo de Manguinhos*, indicating a heterogeneous distribution within the community. The prevalence of moderate/severe FI in *Embratel* slum was nearly double the overall prevalence (28%, 95%CI: 22%-36%) while in *Ex-Combatentes* slum, it was less than half the overall prevalence (4%, 95%CI: 2%-9%).

### Description of the study population

Table 1 describes the study population according to their FI status and shows the absolute and relative frequency of household-level characteristics with household level of FI. Households with no income or income ≤1 minimum wage (MW) exhibited a substantially higher proportion of moderate and severe FI compared to households with higher income. Among households with no income, 10.5% experienced moderate FI and 16.6% severe FI. Conversely, among households with >2 MW, only 3.1% and 2.6% experienced moderate FI or severe FI, respectively. Households receiving *Bolsa Família* benefit showed a higher prevalence of moderate (10.7%) and severe (14.4%) FI compared to non-beneficiaries (moderate FI: 5.2%, severe FI: 6.3%). Similarly, households that received *Auxílio Financeiro Emergencial* were more likely to experience moderate (8.7%) and severe (9.4%) FI than non-beneficiaries (moderate FI: 4.3%, severe FI: 7.1%). The number of children enrolled in *Bolsa Família* also correlated with FI severity: households with three or more children in the program had the highest prevalence of severe FI (19.4%), followed by those with two children (11.6%) and one child (11.5%).

**Table 1:** Study population households’ characteristics according to food insecurity status as measured by the Brazilian Food Insecurity (EBIA) scale, the *Comvida-1* Study, Rio de Janeiro, Brazil, from September 2020 to February 2021 (n = 3,864).

	Food security	Mild FI	Moderate FI	Severe FI	Total	P value
Monthly family income (in minimum wages), n (%)						< 0.001
No income	181 (35.7)	189 (37.3)	53 (10.5)	84 (16.6)	507 (100)	
≤ 1	748 (45.9)	568 (34.8)	135 (8.3)	179 (11)	1630 (100)	
> 1 & ≤ 2	577 (59.4)	308 (31.7)	47 (4.8)	39 (4)	971 (100)	
> 2 & ≤ 3	237 (71)	89 (26.6)	3 (0.9)	5 (1.5)	334 (100)	
> 3	132 (73.3)	42 (23.3)	4 (2.2)	2 (1.1)	180 (100)	
Missing	180 (74.4)	46 (19)	8 (3.3)	8 (3.3)	242 (100)	
*Bolsa Família* benefit, n (%)						< 0.001
No	1690 (58.3)	874 (30.1)	152 (5.2)	184 (6.3)	2900 (100)	
Yes	319 (35.2)	359 (39.7)	97 (10.7)	130 (14.4)	905 (100)	
Missing	46 (78)	9 (15.3)	1 (1.7)	3 (5.1)	59 (100)	
Number of children included in *Bolsa Família*, n (%)						< 0.001
None	1761 (58.1)	910 (30)	157 (5.2)	204 (6.7)	3032 (100)	
One	152 (41.8)	133 (36.5)	37 (10.2)	42 (11.5)	364 (100)	
Two	88 (35.1)	105 (41.8)	29 (11.6)	29 (11.6)	251 (100)	
Three or more	54 (24.9)	94 (43.3)	27 (12.4)	42 (19.4)	217 (100)	
*Auxílio Financeiro Emergencial* benefit, n (%)						< 0.001
No	1209 (63.9)	466 (24.6)	82 (4.3)	134 (7.1)	1891 (100)	
Yes	800 (41.7)	769 (40.1)	167 (8.7)	181 (9.4)	1917 (100)	
Missing	46 (82.1)	7 (12.5)	1 (1.8)	2 (3.6)	56 (100)	
Household size, n (%)						< 0.001
One	227 (60.9)	85 (22.8)	23 (6.2)	38 (10.2)	373 (100)	
Two	469 (60.8)	211 (27.3)	46 (6)	46 (6)	772 (100)	
Three	523 (52.2)	352 (35.2)	63 (6.3)	63 (6.3)	1001 (100)	
Four	432 (52.2)	294 (35.6)	46 (5.6)	55 (6.7)	827 (100)	
Five or more	354 (43)	295 (35.8)	72 (8.7)	102 (12.4)	823 (100)	
Missing	50 (73.5)	5 (7.4)	0 (0)	13 (19.1)	68 (100)	
Received food donation, n (%)						< 0.001
No	1473 (61.4)	634 (26.4)	115 (4.8)	176 (7.3)	2398 (100)	
Yes	535 (37.8)	606 (42.9)	135 (9.5)	138 (9.8)	1414 (100)	
Missing	47 (90.4)	2 (3.8)	0 (0)	3 (5.8)	52 (100)	
Working hours reduced, n (%)						< 0.001
No	1603 (55.8)	859 (29.9)	168 (5.9)	241 (8.4)	2871 (100)	
Yes	404 (43)	380 (40.5)	82 (8.7)	73 (7.8)	939 (100)	
Missing	48 (88.9)	3 (5.6)	0 (0)	3 (5.6)	54 (100)	
Lost job or income source, n (%)						< 0.001
No	1751 (56.9)	927 (30.1)	171 (5.6)	226 (7.3)	3075 (100)	
Yes	256 (34.7)	314 (42.6)	79 (10.7)	88 (11.9)	737 (100)	
Missing	48 (92.3)	1 (1.9)	0 (0)	3 (5.8)	52 (100)	
Family income varied, n (%)						< 0.001
No change	1280 (67.8)	465 (24.6)	56 (3)	88 (4.7)	1889 (100)	
Decreased	582 (34.5)	717 (42.5)	179 (10.6)	209 (12.4)	1687 (100)	
Increased	46 (46.9)	38 (38.8)	8 (8.2)	6 (6.1)	98 (100)	
Number of bedrooms, n (%)						0.002
One	667 (50.9)	422 (32.2)	106 (8.1)	116 (8.8)	1311 (100)	
Two	1114 (53.2)	678 (32.4)	123 (5.9)	178 (8.5)	2093 (100)	
Three or more	274 (59.6)	142 (30.9)	21 (4.6)	23 (5)	460 (100)	
Persons per bedroom, n (%)						< 0.001
≤ 1	529 (61.9)	209 (24.5)	49 (5.7)	67 (7.8)	854 (100)	
1.1-1.5	425 (57.6)	242 (32.8)	37 (5)	34 (4.6)	738 (100)	
1.6-3.0	909 (50.3)	637 (35.3)	111 (6.1)	149 (8.3)	1806 (100)	
> 3.0	142 (35.7)	149 (37.4)	53 (13.3)	54 (13.6)	398 (100)	
Any children (1–9 years) in the household? n (%)						< 0.001
No	1473 (58.2)	722 (28.5)	148 (5.8)	190 (7.5)	2533 (100)	
Yes	582 (43.7)	520 (39.1)	102 (7.7)	127 (9.5)	1331 (100)	
Any adolescents (10–17 years) in the household? n (%)						< 0.001
No	1436 (58)	727 (29.4)	134 (5.4)	177 (7.2)	2474 (100)	
Yes	619 (44.5)	515 (37.1)	116 (8.3)	140 (10.1)	1390 (100)	
Any elders (≥60 years) in the household? n (%)						< 0.001
No	1205 (48.2)	882 (35.3)	177 (7.1)	237 (9.5)	2501 (100)	
Yes	850 (62.4)	360 (26.4)	73 (5.4)	80 (5.9)	1363 (100)	

FI: food insecurity.

Receiving food donations, reduced working hours, and job or income losses were significantly more prevalent among food-insecure households (9.5% of households that received food donations experienced moderate FI and 9.8% severe FI). Among households that reported reduced working hours, 8.7% experienced moderate FI and 7.8% severe FI. Similarly, among households affected by job or income losses, 10.7% experienced moderate FI and 11.9% severe FI. Among households with decreased income, 10.6% experienced moderate FI and 12.4% experienced severe FI.

Household size was associated with FI. Larger households (five or more members) were disproportionately affected by moderate (8.7%) and severe (12.4%) FI, whereas smaller households (one or two members) were more frequently food secure. The presence of children (age 1–9 years) and adolescents (age 10–17 years) in the household was also associated with higher prevalence of FI (7.7% and 9.5% of the households with children experienced moderate and severe FI, respectively; whereas 8.3% and 10.1 of households with adolescents experienced moderate and severe FI). Households with higher density (>3.0 persons per bedroom) exhibited higher prevalence of moderate (13.3%) and severe (13.6%) FI, while among households with lower densities (≤1.0 persons per bedroom) the prevalence of moderate (5.7%) and severe (7.8%) FI was lower.

### Multiple correspondence analysis

**Fig 3:**
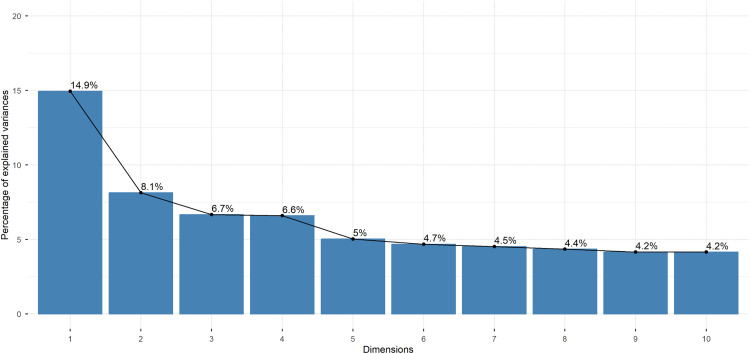
Scree plot including the top 10 MCA dimensions, the *Comvida-1* Study, Rio de Janeiro, Brazil, from September 2020 to February 2021 (n = 3,864). The scree plot displays the percentage variance explained by each dimension, ordered from highest (Dimension 1) to lowest (Dimension 10). The x‑axis represents the dimension number (1 to 10); the y‑axis shows the percentage of variance explained by that dimension. The “elbow” is the point where the curve of the descending bar heights bends and begins to flatten. Dimensions before this point (Dimensions 1 to 5) are considered meaningful and were retained for interpretation, as they represent the most important underlying patterns in the data. Dimensions after the elbow (Dimension 6 onwards) are considered to represent noise or residual variance and therefore were discarded.

**Fig 4:**
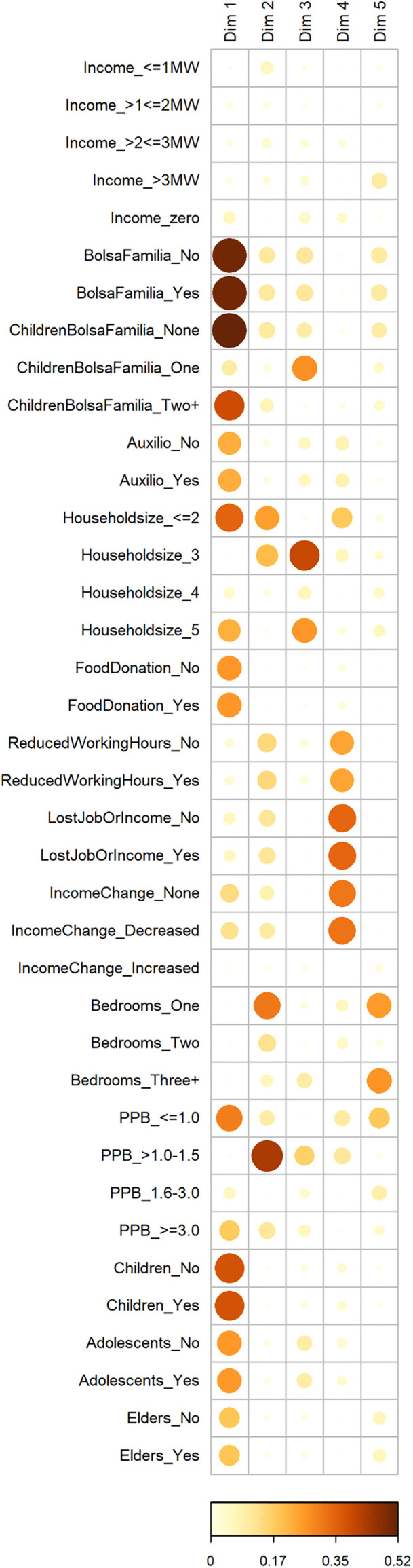
Degree of association between variable categories and the MCA dimensions (only showing dimension 1 through 5), the *Comvida-1* Study, Rio de Janeiro, Brazil, from September 2020 to February 2021 (n = 3,864). Heatmap displaying variable categories’ contributions to each MCA dimension, with dot size and color (yellow = lower contribution, dark red = higher contribution) representing contribution magnitude. MW: minimum wage; PPB: persons per bedroom.

Fig 3 illustrates the percentages of variance (total inertia) explained by each MCA dimension showing that the dimensions five and above consistently explain less than 5% of the total variance. Dimensions 1–5 capture the majority of the meaningful variation in the data, with subsequent dimensions likely representing noise rather than systematic patterns. Fig 4 shows the degree of association between variables’ categories and the first five MCA dimensions. Multiple categories were highlighted for dimensions one and four whereas dimensions two and three seem mostly associated with one category (PPB between 1.1-1.5 and household size of three, respectively).

### Multilevel logistic regression models

An initial multilevel logistic regression model included only a random intercept for community-level clustering (slum) and the outcome. The variance for the random intercept was 0.1183, and the intraclass correlation coefficient was 0.034, indicating that about 3.4% of the total variance in FI is attributable to differences between slums. Given the statistical significance of the random effect, we proceeded to include a slum-level factor in the regression model.

Multilevel logistic regression models were utilized to assess the association of the identified MCA dimensions with moderate/severe FI, while accounting for the spatial clustering of participants within the 16 slums of *Complexo de Manguinhos*. The performance metrics of the regression models is summarized in Table 2. Model 4, incorporating dimension 1 and dimension 4 as covariates along with the slum-level factor, showed the best performance with the highest accuracy, sensitivity, specificity and AUC.

**Table 2:** Multilevel logistic regression models’ performance metrics and final model’s (Model 4) estimated adjusted odds ratios (95% confidence interval), outcome moderate/severe food insecurity (measured by the Brazilian Food Insecurity Scale, EBIA), the *Comvida-1* Study, Rio de Janeiro, Brazil, from September 2020 to February 2021 (n = 3,864).

Model	Covariates	Accuracy	Sensitivity	Specificity	AUC
Model 1	Dim1 + Dim2 + Dim3 + Dim4 + Dim5 +(1|slum)	0.864655	0.99503	0.012987	0.71355
Model 2	Dim1 + Dim2 + Dim3 + Dim4+(1|slum)	0.864655	0.99503	0.012987	0.71435
Model 3	Dim1 + Dim2 + Dim4+(1|slum)	0.866379	0.997018	0.012987	0.713679
Model 4	Dim1 + Dim4+(1|slum)	0.868103	0.999006	0.012987	0.716816
Model 5	Dim1+ (1|slum)	0.8672	1	0	0.6743
**Model 4 Coefficients**					
**Fixed-effects**					
	**Adjusted Odds Ratio (95%CI)**				
Dim 1	2.72 (2.19-3.38)				
Dim 4	3.32 (2.42-4.55)				
**Random effects**					
τ00	0.12				
ICC	0.03				
Marginal R^2^/ Conditional R^2^: 0.11/ 0.141					

AUC: area under the receiver operator curve; dim: dimension; CI: confidence interval; τ00: variance of the random intercepts; ICC: Intraclass Correlation Coefficient.

The coefficients of dimension 1 and dimension 4 were significant (p < 0.001). One-unit increase in dimension 1 increased the odds of moderate/severe FI by 2.72 (95% CI: 2.19-3.38), meaning 172% higher odds of moderate/severe FI for each unit increase in dim1. One unit increase in dimension 4 increased the odds of moderate/severe FI by 3.32 (95% CI: 2.42-4.55), indicating 232% higher odds of moderate/severe FI for each unit increase.

### Interpretation of MCA dimensions associated with food insecurity

**Fig 5:**
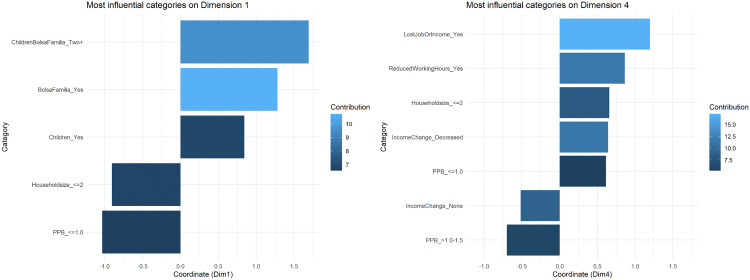
Contribution and coordinates of the most influential categories (contribution ≥ 5%) on Dimension 1 and Dimension 4, the *Comvida-1* Study, Rio de Janeiro, Brazil, from September 2020 to February 2021 (n = 3,864). Left panel shows Dimension 1; right panel shows Dimension 4; X-axis shows the categories’ coordinate values on each dimension, with greater distance from zero indicating stronger association with the dimension. Blue color intensity also represents contribution magnitude (darker = lower contribution, lighter = higher contribution). PPB: persons per bedroom.

Dimension 1 shows a strong representation of socioeconomic vulnerability and reliance on governmental support (Fig 5). Categories with the highest contributions to this dimension included: *Bolsa Família* benefit (10.73%), households with two or more children enrolled in *Bolsa Família* (9.56%), and presence of children in the household (6.84%). The categories’ coordinates in this dimension reveal a clear opposition between households receiving *Bolsa Família* benefit with multiple children (positive coordinates) and smaller and with low density households (household size ≤2 and PPB ≤ 1.0) (negative coordinates).

**Fig 6:**
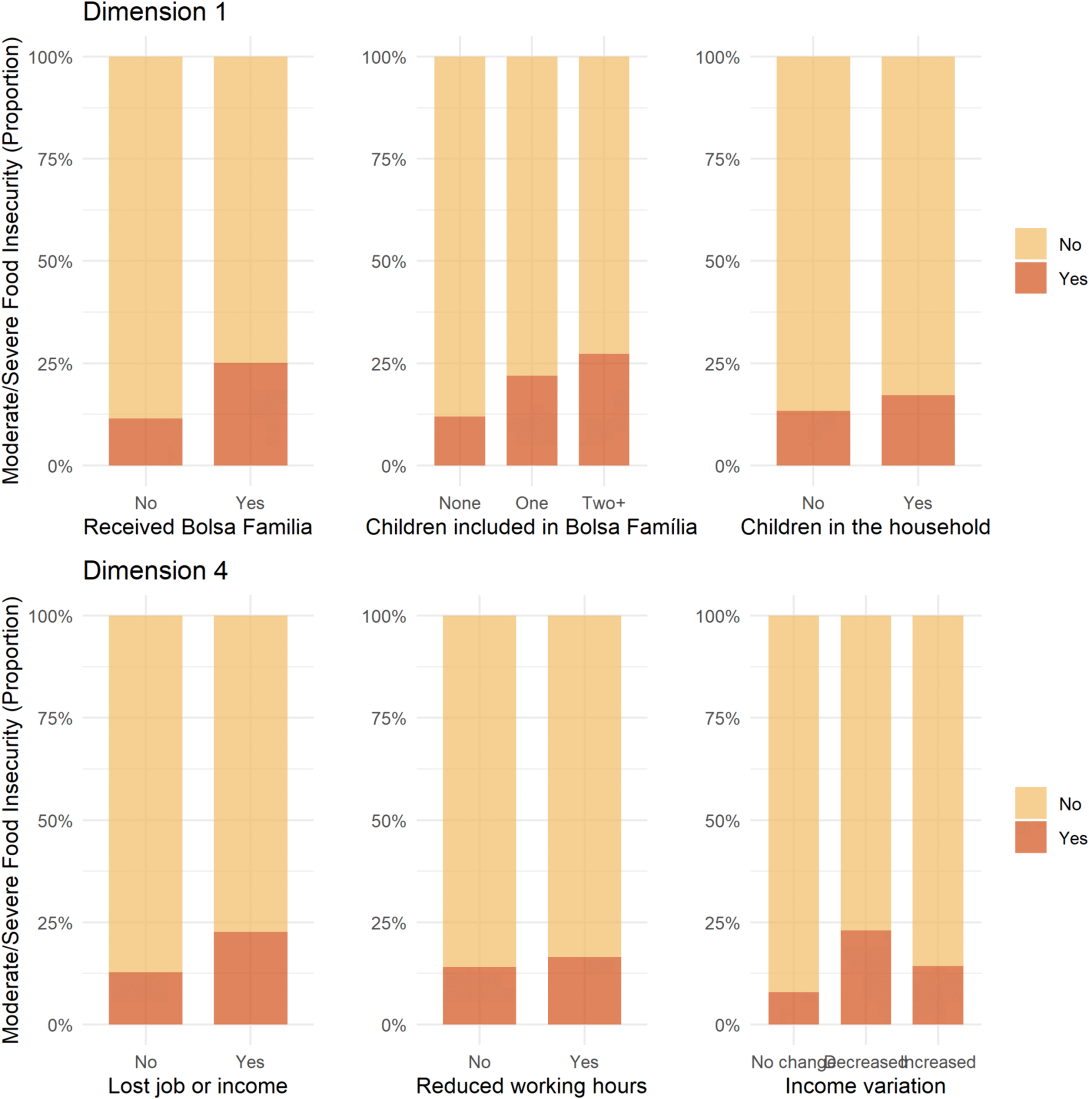
Prevalence of moderate/severe food insecurity (measured by the Brazilian Food Insecurity Scale, EBIA) across the most influential variables in dimension 1 and dimension 4, the *Comvida-1* Study, Rio de Janeiro, Brazil, from September 2020 to February 2021 (n = 3,864). The bar plot shows the prevalence of moderate/severe food insecurity stratified by categories of the most influential variables from Dimensions 1 and 4 of the Multiple Correspondence Analysis. The x-axis shows the categories of the variables; the y-axis shows the proportion of moderate/severe FI within each category.

Dimension 4 was strongly associated with sudden income loss and labor market disruptions experienced during the COVID-19 pandemic (Fig 5). Variables with the highest contributions to this dimension included: lost job or income (17.24%), reduced working hours (11.44%), and reduced income (11.30%). The categories’ coordinates in this dimension show that categories related to pandemic-related economic hardship (positive coordinates) are positioned opposite to households with stable incomes and moderate household density (negative coordinates). As such, this dimension seems to reflect economic precarity triggered by the pandemic, including those who experienced direct impacts like job loss, reduced work hours, and explicit income drops, in addition to households that, despite no income change, likely remained in precarious low-wage situations. Unlike dimension one, variables related to chronic poverty or welfare dependence contributed minimally to dimension four. The prevalence of severe/moderate FI across the most influential categories in dimensions 1 and 4 is shown in Fig 6.

## Discussion

Our study revealed a high prevalence of moderate/severe FI among the study population (14.7%, 95%CI 13.5-15.9). This proportion is higher than the 9% nationwide prevalence estimated for Brazil in a national population-based survey carried out in December 2020 [13]. The high prevalence of moderate/severe FI observed in our study is likely a result of the pronounced socioeconomic vulnerability within *Complexo de Manguinhos*. Our study population exhibited several markers of socioeconomic vulnerability: 80% had a monthly family income below two minimum wages, 42% of households had four or more residents, one-third had children aged 1–9 years, and one-third had elderly residents (aged ≥60 years). The critical role of socioeconomic vulnerability is further highlighted when contrasting our findings with other Brazilian studies. For instance, a study carried out in Fortaleza (capital of Ceara state located in the northeast region of Brazil) among families with infants born during the pandemic (July and August 2020) also reported a high prevalence of moderate/severe FI (20%) [30], where 90% of the households had a family income below two minimum wages and 60% had four or more residents. Conversely, a populational-based survey conducted in two cities in Minas Gerais state (southeast region of Brazil) between October and December 2020 reported a much lower prevalence of moderate/severe food insecurity (3%) but also a much smaller proportion (41%) of the families had a family income below two minimum wages [31]. Corroborating these findings, a meta-analysis of Latin American studies identified low socioeconomic status, households with four or more residents and the presence of children (<10 years) and elderly members (>60 years) as factors associated with higher odds of food insecurity during COVID-19 pandemic [32].

Our analysis identified households receiving *Bolsa Família* benefit (10.73% contribution) and those with two or more children enrolled in the program (9.56% contribution) as the strongest contributors to Dimension 1, and that Dimension 1 was a significant predictor of moderate/severe FI. These results corroborate the established association between FI and economic fragility among social welfare-dependent populations. These findings are consistent with those from national PENSSAN surveys and research conducted with families with infants in Fortaleza, mentioned previously [12,13,30]. Importantly, while *Bolsa Família* remains essential for poverty reduction, our findings suggest its monetary value during the COVID-19 pandemic was insufficient to fully protect beneficiaries against FI, particularly in contexts of extreme socioeconomic vulnerability such as the *Complexo de Manguinhos*.

In contrast to the chronic poverty patterns captured by Dimension 1, Dimension 4 captured aspects related to sudden income loss, including job loss, reduced work hours, and decreased earnings. Notably, *Bolsa Família* showed minimal contribution to this dimension, underscoring Dimension’s 4 distinct nature as representing pandemic-induced labor market disruptions rather than chronic poverty. Our results showed that Dimension 4 was an even stronger predictor of moderate/severe FI. This finding demonstrates that sudden income loss immediately compromised food access even for households not previously classified as chronically poor. Furthermore, the higher odds ratio observed for Dimension 4 compared to Dimension 1 suggest that these transient economic shocks exerted a more pronounced immediate effect on FI than longstanding socioeconomic vulnerability. These results align with PENSSAN survey findings, which similarly identified income loss and unemployment as significant determinants of FI during the pandemic period [12,13].

FI increased in Brazil during the COVID-19 pandemic [11], a trend that aligns with the broader Latin American experience. In Chile, a population-based study documented a significant increase in FI between 2017–2020, which disproportionately affected households with children and adolescents [33]. Furthermore, unemployment was a consistent driver of higher FI, both before and during the pandemic [33]. In a national Mexican study, FI also rose monthly during the mandated lockdown, with a similar disproportional impact on households with children [34].

Existing research has consistently documented FI disparities between neighborhoods of different socioeconomic strata, linking disadvantaged areas to less access to healthy, affordable food choices and an excess of fast-food restaurants [35]. Moving beyond this macro-level view, our study reveals significant disparities within a vulnerable setting. We found that the prevalence of moderate/severe FI varied across the 16 slums of *Complexo de Manguinhos*, ranging from 4% (*Ex-Combatentes*) to 28% (*Embratel*). This seven-fold difference indicates considerable spatial clustering of FI even within a highly vulnerable urban community. Multilevel analysis confirmed that 3.4% of the total variance in FI stemmed from between-slum differences. While this intraclass correlation might appear modest, it reflects meaningful community-level clustering likely associated with differential access to resources, infrastructure, and social networks [35]. These results underscore the importance of disaggregated analysis in social epidemiology, as community-level averages may mask critical pockets of heightened risk that require targeted interventions. Understanding the specific community-level factors driving these disparities among slums could inform more precise and effective food security interventions within similar urban contexts.

FI is driven by a complex interplay of factors, ranging from individual- and household-level drivers to neighborhood conditions and structural determinants like social, economic and political context [35]. Individuals and households experiencing poverty, material hardship, or unemployment are at greater risk of FI. Moreover, these vulnerable populations frequently reside in neighborhoods characterized by low access to food and healthcare, compounded by poor housing conditions and inadequate sanitation. Systemic racism and other discriminatory forces fuel this cycle, disproportionately burdening racial/ethnic marginalized populations, gender and sexual minorities and people with disabilities [35,36]. The COVID-19 pandemic and the economic shock have also disproportionately affected these populations, synergistically interacting with pre-existing vulnerabilities. Our results suggest the existence of two distinct but complementary pathways, chronic poverty and acute economic shock, that likely overlap emphasizing the complexity of interventions strategies to address FI.

Future pandemic preparedness requires anticipating the limitations of existing social protection systems during health emergencies. Our findings demonstrate that even successful welfare programs like *Bolsa Família*, while important for poverty alleviation, may prove insufficient during crises when economic shocks intensify existing vulnerabilities [37,38]. Additionally, given that both chronic structural inequities and acute economic disruptions contribute to FI through distinct pathways, effective pandemic response requires coordinated interventions that can simultaneously address immediate needs while strengthening long-term resilience. Despite the implementation of a large-scale emergency federal financial assistance program, *Auxílio Financeiro Emergencial*, which allocated over 288 billion BRL (approximately 55 billion USD) during COVID-19 [39], our results suggest that its impact on moderate/severe FI was probably modest. This highlights the need for differentiated and complementary emergency interventions: enhanced benefits for chronically poor households already in *Bolsa Familia* (particularly households with children), and new income replacement programs for those experiencing sudden job or income losses who fall outside existing social protection coverage. Investment in local-level vulnerability mapping would enable more targeted emergency responses, allowing policymakers to identify and prioritize communities at highest vulnerability before crises occur.

This study has both limitations and strengths that warrant consideration. Our cross-sectional design limits the ability to establish the temporal sequence between exposure variables and the study outcome as both were measured at the same time. We handled missing data among variables included in the MCA by mode imputation. While we acknowledge that more robust methods exist, like Multiple Imputation by Chained Equations (MICE) [40], we made a context-specific decision taking into account the low-frequency of missingness, the nature of the missing values and our research question. We justify this choice because for every affected variable, the imputed mode represented the more conservative category—specifically, the absence of a socioeconomic shock. For instance, the imputed values were ‘no’ for receiving Bolsa Familia, ‘no’ for receiving food donation, ‘no’ for job or income loss, and ‘no’ for reduced work hours. Similarly, for household size and monthly family income, the imputed modes were towards the median. We reason that this approach was more likely to attenuate any association (i.e., bias towards the null) between these socioeconomic indicators and FI than to inflate them. We documented differences in the prevalence of moderate/severe FI across the 16 slums in *Complexo the Manguinhos*. However, micro-level characterization of each favela, including their public facilities, community support networks, local religious and non-religious organizations, and food environment would strengthen our analysis. Our study population was probabilistically sampled from individuals residing in a complex of slums in Rio de Janeiro and may not be generalizable to other populations. Yet, our findings might be common to other marginalized urban populations in Brazil and other countries in Latin America. A strength of our study was the methodological robustness of our analyses, specifically the use of MCA and multilevel regression modeling to account for complex data structures and community-level clustering.

## Conclusion

Our study demonstrates that both chronic vulnerability (Dimension 1) and COVID-19 economic shocks (Dimension 4) were significant predictors of moderate/severe FI through distinct pathways: Dimension 1 captured structural inequities tied to long-term poverty, while Dimension 4 reflected the acute effects of sudden economic instability. Community-level differences, though modest, further nuanced these findings. Policymakers should address FI through layered interventions—bolstering social protections for chronic vulnerability (e.g., adjusting of *Bolsa Familia* benefit, particularly for families with children) while implementing rapid-response safeguards for economic crises (e.g., emergency cash transfers and job retention programs).

## Supporting information

S1 Table Comparison between the study population included in this secondary analysis to those enrolled in the Comvida-1 study but excluded from this secondary analysis, the Comvida-1 Study, Rio de Janeiro, Brazil, from September 2020 to February 2021 (n = 4,033). (DOCX)

S2 Table The 14 items of the Brazilian Food Insecurity Scale (Escala Brasileira de Insegurança Alimentar, EBIA) for classifying household food security. (DOCX)
