# Supplementary material for: Food insecurity and associated factors during the COVID-19 pandemic in a vulnerable population in Rio de Janeiro: A primary care registry-based survey
Source: PLOS Glob Public Health. 2025 Dec 15;5(12):e0005406. doi: 10.1371/journal.pgph.0005406 (PMC12704868; doi:10.1371/journal.pgph.0005406)
Supplement: S1 Table — (DOCX) [file pgph.0005406.s001.docx]

**S1 Table. Comparison between the study population included in this secondary analysis to those enrolled in the Comvida-1 study but excluded from this secondary analysis, the Comvida-1 Study, Rio de Janeiro, Brazil, from September 2020 to February 2021 (n=4,033).**

|  | Included in the analysis | Excluded from the analysis | p-value |
| --- | --- | --- | --- |
|  | N = 3864 | N = 169 |  |
| Age in years, median (IQR) | 39.8 (21.8,57.7) | 38 (21.7,57.6) | 0.481 |
| Age in years, categories, n (%) | |  | 0.965 |
| 1-4 | 107 (2.8) | 5 (3) |  |
| 5-9 | 188 (4.9) | 10 (5.9) |  |
| 10-19 | 551 (14.3) | 25 (14.8) |  |
| 20-29 | 575 (14.9) | 26 (15.4) |  |
| 30-39 | 523 (13.5) | 23 (13.6) |  |
| 40-49 | 532 (13.8) | 27 (16) |  |
| 50-59 | 550 (14.2) | 20 (11.8) |  |
| 60-69 | 507 (13.1) | 19 (11.2) |  |
| 70-79 | 247 (6.4) | 12 (7.1) |  |
| 80+ | 84 (2.2) | 2 (1.2) |  |
| Sex |  |  | 0.387 |
| Male | 1523 (39.4) | 61 (36.1) |  |
| Female | 2341 (60.6) | 108 (63.9) |  |

Percentage comparisons between those included and excluded from the analysis were made using the Chi-squared test, medians comparison was made using the Kruskal-Wallis test.
