# Supplementary material for: Food insecurity and associated factors during the COVID-19 pandemic in a vulnerable population in Rio de Janeiro: A primary care registry-based survey
Source: PLOS Glob Public Health. 2025 Dec 15;5(12):e0005406. doi: 10.1371/journal.pgph.0005406 (PMC12704868; doi:10.1371/journal.pgph.0005406)
Supplement: S2 Table — (DOCX) [file pgph.0005406.s002.docx]

**S2 Table. The 14 items of the Brazilian Food Insecurity Scale (*Escala Brasileira de Insegurança Alimentar*, EBIA) for classifying household food security.**

| # | Content |
| --- | --- |
| 1 | Did household members (≥18 years) worry that they would run out of food before being able to buy or receive more food? |
| 2 | Did household members (≥18 years) run out of food before having money to buy more? |
| 3 | Did household members (≥18 years) run out of money to have a healthy and varied diet? |
| 4 | Did household members (≥18 years) eat only a few kinds of foods they still had because they had run out of money? |
| 5 | Did any household member (≥18 years) skip a meal because there was no money to buy food? |
| 6 | Did any household member (≥18 years) eat less than what he/she felt he/she should because there was no money to buy food? |
| 7 | Did any household member (≥18 years) feel hungry but did not eat because there was no money to buy food? |
| 8 | Did any household member (≥18 years) have just one meal a day or didn’t eat for a whole day because there was no money to buy food? |
| 9 | Did any household member aged less than 18 years stop having a healthy and varied diet because there was no money to buy food? |
| 10 | Did any household member aged less than 18 years not have enough to eat because there was no money to buy food? |
| 11 | Did any person in your household aged less than 18 years have to reduce the size of meals because there was no money to buy food? |
| 12 | Did any household member aged less than 18 years skip a meal because there was no money to buy food? |
| 13 | Did any household member aged less than 18 years feel hungry but could not eat because there was no money to buy more food? |
| 14 | Did any household member aged less than 18 years have just one meal a day or went without eating for a whole day because there was no money to buy food? |

All items have the same response options (“yes”, “no”, “don’t know”) and refer to the period of the COVID-19 pandemic. Each affirmative answer (“yes” response) to any of the 14 items receives one point, while "no" and "don't know" responses receive zero points, resulting in a score ranging from 0 to 14 points. This score enables the classification of households into food secure (0 points) and food insecure (≥ 1 point). Moreover, it classifies households into three levels of FI: mild (1-5 points), moderate (6-9 points) and severe (10-14 points).
